# Sociomarkers and biomarkers: predictive modeling in identifying pediatric asthma patients at risk of hospital revisits

**DOI:** 10.1038/s41746-018-0056-y

**Published:** 2018-10-02

**Authors:** Eun Kyong Shin, Ruhi Mahajan, Oguz Akbilgic, Arash Shaban-Nejad

**Affiliations:** 1 Department of Pediatrics, University of Tennessee Health Science Center – Oak Ridge National Laboratory- (UTHSC-ORNL), Center for Biomedical Informatics, Memphis, TN USA; 2 Department of Preventive Medicine, UTHSC, Memphis, TN USA

**Keywords:** Risk factors, Population screening

## Abstract

The importance of social components of health has been emphasized both in epidemiology and public health. This paper highlights the significant impact of social components on health outcomes in a novel way. Introducing the concept of sociomarkers, which are measurable indicators of social conditions in which a patient is embedded, we employed a machine learning approach that uses both biomarkers and sociomarkers to identify asthma patients at risk of a hospital revisit after an initial visit with an accuracy of 66%. The analysis has been performed over an integrated dataset consisting of individual-level patient information such as gender, race, insurance type, and age, along with ZIP code-level sociomarkers such as poverty level, blight prevalence, and housing quality. Using this uniquely integrated database, we then compare the traditional biomarker-based risk model and the sociomarker-based risk model. A biomarker-based predictive model yields an accuracy of 65% and the sociomarker-based model predicts with an accuracy of 61%. Without knowing specific symptom-related features, the sociomarker-based model can correctly predict two out of three patients at risk. We systematically show that sociomarkers play an important role in predicting health outcomes at the individual level in pediatric asthma cases. Additionally, by merging multiple data sources with detailed neighborhood-level data, we directly measure the importance of residential conditions for predicting individual health outcomes.

## Introduction

Health is a social as much as a biological matter, not simply because of the infectious nature of some diseases, but also because social conditions heavily influence health outcomes at the individual level.^1–4^ A vast body of knowledge on the link between health and social factors has been accumulated in social epidemiology.^5–11^ The importance of the social factors is increasingly acknowledged as one of the most critical health determinants, and the non-genetic components are estimated as a significant (70%) contributor to individuals’ health.^2,3,5,12^ Among various social factors, neighborhood level has been suggested as a key parameter for explaining how social inequalities are engraved into health inequalities.^13–18^

Despite the importance of the social determinants of health, the application of social factors for the clinical decision-making is still in its nascent stage. There are limited social features available in the public health surveillance data sets. The continuing concerns regarding the inaccurate estimation of any effect at the aggregated level based on individual-level data, which is referred to as ecological fallacy, hinder further aggressive empirical analyses.^19,20^ Recent methodological development in multi-level modeling provides some important leverage regarding how to combine individual-level data with macro-level data.^10,21,22^ Still, traditional statistics are sensitive to data sampling. The even distribution of each observed category is essential to arrive at statistically significant findings. The scarcity of data availability and methodological restrictions make this line of research extremely challenging.

Avoiding these methodological problems in enhancing our understanding of social epidemiology, we assess how social features perform in identifying patients at risk of hospital revisit due to asthma within a year, in comparison with the traditional biomarkers.^23^ We introduce the concept of sociomarkers, which are measurable indicators of social conditions in which a patient is embedded and is exposed to, being analogous to a biomarker indicating the severity or presence of some disease state. Sociomarkers can help medical practitioners and researchers to reliably identify high-risk individuals, who are more likely to revisit the hospital with the asthma-related case, in a timely manner for efficient health surveillance. The clinical condition under consideration for the present study is pediatric asthma, which is aptly pertinent to the question for three reasons. First, asthma is one of the most common chronic childhood diseases in the United States.^24,25^ Second, in addition to its pervasiveness, its sensitivity to the environment makes the subject particularly pertinent to the question under investigation.^22,26,27^ Lastly, most asthma-related hospital visits can be prevented by appropriate preventive care.^28^

In this paper, by linking the electronic health record data repository and external housing and neighborhood quality datasets provided by our private sector partner, we employ a machine learning-based classification model to test whether sociomarkers can be used as indicators to identify pediatric asthma patients at risk of hospital revisits. Hospital readmissions for pediatric asthma patients have been extensively investigated in the literature.^28–34^ The risk of hospital readmission for pediatric asthma patients exponentially increases with its repetition. African American children with low economic status are at higher risk of hospital readmission.^29^ Also, age^28,32^ and gender^35^ of patients are important factors in explaining the risk of readmission to hospital in asthma cases. In addition to these demographic characteristics, social characteristics have been known to play important role in pediatric asthma readmission: patients who reside in a lower economic status neighborhood and those who are covered by Medicaid have a higher risk of hospital readmission.^22,29^

**Fig. 1:**
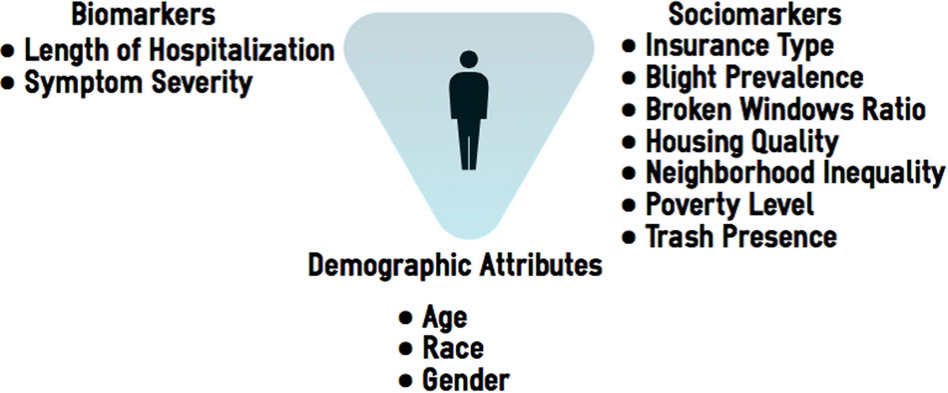
Analytic framework: Sociomarkers and biomarkers

We train the machine learning-based classification models with three different sets of features: demographic attributes of a patient (age, race, and gender), biomarkers representing the patient’s medical conditions (critical symptom-related features such as length of hospital stay and symptom severity,^36,37^) and sociomarkers. Then we compare their performance to evaluate the validity and relevance of sociomarkers in predicting whether at-risk pediatric asthma patients will revisit the hospitals. For sociomarkers, building on the existing literature, we expand the social characteristic to urban residential built environments as well, capturing environmental and social characteristics related to the patient’s residential area and the social economic status of patients measured by the insurance type. All above-mentioned features can be harvested through the routine medical procedures. We link the patient data through the ZIP code with existing social and environmental datasets. With these three types of patient data, as presented in Fig. 1, we run three sets of predictive models. In the first model (referred to as Model 1, or all-inclusive model) we include all three types of features to gauge how a machine learning-based classification model performs overall in predicting whether at-risk patients will revisit the hospitals with the primarily asthma-related cases. In the second model, we only use the patient-level features of demographics and biomarkers (referred to as Model 2, biomarker-based model), and in the last model (referred to as Model 3, sociomarker-based model), we only consider demographic information and sociomarkers as predicting features.Please note that all three models include the demographics as the base components. By comparing the performance of machine learning-based classification models, we empirically assess the importance and significance of social components in health outcomes at the individual level. However, by comparing Model 2 and Model 3, we can empirically test the contribution of social components versus more traditional symptom-related features in the prediction of health outcomes.

## Results

As shown in Table 1, we have analyzed 12 features related to demographic attributes, biomarkers, and sociomarkers of patients, implementing a random forest (RF) classification^38^ model to identify subjects at risk of a second hospital visit within one year. To ensure generalizability of the model, we trained our random forest model on the randomly selected 80% training data and tested the model on the remaining 20% of data. Further, to avoid overfitting in the model, we implemented five-fold cross-validation on the training dataset. In Model 1, in which we have used all available features (demographics, biomarkers, and sociomarkers), we obtained an average classification accuracy of 66.11% for the training set and 66.05% for the test set, as presented in Table 2. To evaluate the performance of the proposed model, we also calculated specificity and sensitivity from the confusion matrix. We obtained a specificity and sensitivity of 67.67 and 64.82% from the five-fold cross-validated training set and 67.63 and 64.82% from the test set, respectively. We can see that the model performs stably given the similarity across the test set and the training set. Overall, this all-inclusive model can predict the pediatric asthma patient at risk of hospital revisit with an accuracy of 66.05%.

**Table 1:** Variables and operationalization

	Operationalization	*N*(%)/Mean (SD)
Revisit dummy	0: No revisit in 2016	2855 (77.62)
	1: Revisit in 2016	823 (22.38)
Gender	0: Female	1442 (39.21)
	1: Male	2236 (60.79)
Race	0: White	389 (10.58)
	1: 1: African American	3289 (89.42)
Age	Age of a patient in years	7.422 (4.66)
Length of hospitalization	Days of hospitalization	.90 (1.43)
Symptom severity	Severity of pediatric asthma (ICD 10 codes)	1.57 (0.60)
Insurance type	0: Non-medicaid Patient	533 (14.49)
	1: Medicaid Patient	3145 (85.51)
Blight prevalence	The ratio of the unoccupied properties within a ZIP code area	.048 (.024)
Housing quality	Mean of ratings of the property qualities located within a ZIP code area (1: Excellent and 5: Severely dilapidated)	1.90 (.283)
Neighborhood inequality	Standard deviation of housing quality data within a ZIP code area	.80(.18)
Poverty level	Percentage of individuals under the federal poverty level within a ZIP code area	.31(.11)
Trash presence	The ratio of the properties with dumped trash within a ZIP code area	.0050(.0047)

**Table 2:** Classification statistics (in %) for each model with RF and SVM techniques

		Test set			Training set		
		Acc.	Spec.	Sens.	Acc.	Spec.	Sens.
RF	Model 1	66.05	67.63	64.82	66.11	67.67	64.82
	Model 2	65.39	67.11	64.07	65.48	67.12	64.14
	Model 3	61.17	62.59	60.11	61.28	62.70	60.16
SVM	Model 1	62.10	62.00	62.32	62.21	62.10	62.35
	Model 2	59.58	59.89	59.41	59.70	59.96	59.48
	Model 3	57.83	59.07	56.98	57.97	59.17	57.08

By using only demographics and biomarkers attributes in Model 2, we achieved a little lower accuracy of 65.48 and 65.39% from the five-fold cross-validated training set and test set, respectively. The specificity and sensitivity for the training and test sets were determined as 67.12, 64.14, 67.11, and 64.07%, respectively. This suggests that symptom-related features can identify the patient at risk with an accuracy of 65%. With Model 3, based on demographics and sociomarkers, we obtained an average classification accuracy of 61.28% from the cross-validated training set and 61.17% from the test set, respectively. The specificity and sensitivity for the training and test sets are 62.70, 60.16, 62.59, and 60.11%, respectively. Interestingly, without using any symptom-related predictors, simple information gathered from the ZIP code level and demographic characteristics of a patient still allow us to predict which patients will revisit the hospital with 61% accuracy and the results are stable with test data as well. We also implemented a secondary cross-validation for Model 3 by making sure that the training and testing datasets are extracted from different ZIP codes to evaluate whether our models would be valid for data coming from different neighborhoods. Our secondary cross-validation yielded 56.66% accuracy on the training data and 66.60% on the test data. Our results showed that the models we develop can be generalized to the other neighborhoods considered in this study. In this supplementary analysis, we note that the testing performance is better than the training performance. This unknown fit, where validation error is low and training error is relatively high,^39^ is may be due to the design of the ZIP code based cross-validation where the distribution of the total sample size and the distribution of Class0- Class1 cases across ZIP code areas significantly varies. However, deeper analysis on a larger dataset may be required to understand the reasons of unknown fit in this problem.

We also evaluated Support Vector Machine (SVM);^40^ however, the random forest classifier yielded the best classification performance on the training and test set.^41–43^ It can be observed from Table 2 that the proposed model does not over-fit and provides similar results for training and test datasets. The classification results from the SVM classifier are also presented in Table 2 and it can be noticed that the SVM classifier did not perform as much as the random forest classifier. The average accuracy, specificity, and sensitivity for the three models using the test set were found to be 62.1, 59.58, and 57.83%, respectively. Interestingly, the contribution margin of the sociomarkers are larger in this case as the difference of the means of the accuracy of Model 1 and Model 2 is 2.52 (compared to 0.65 in the random forest case).

Furthermore, to evaluate the null hypothesis that the mean accuracy obtained from each model, we conducted a two-sample t-test on the accuracies obtained from 1000 iterations of the test set from each model. As shown in Table 3, there are statistically significant differences between all models. The difference between Model 1 and Model 2 can show the statistically significant contribution of sociomarkers in predictive modeling. With the random forest classifier, the differences between the two groups are statistically significant at 0.001. The mean difference is 0.65 and the 95% confidence interval of the mean difference value of accuracies was found to be from 0.47 to 0.84. With the SVM classifier, comparing the difference between Model 1 and Model 2, the 1000 SVM results of Model 1 are statistically different from 1000 SVM results of Model 2. The mean difference is 2.51 and the 95% confidence interval of the mean difference value of accuracies was found to be from 2.31 to 2.70. Overall, the contribution of the sociomarkers is larger in the results using the SVM classifier.

**Table 3:** Two-tailed *t*-test results to compare accuracies of models

	Model 1 vs. Model 2	Model 1 vs. Model 3	Model 2 vs. Model 3
RF	0.65 ***	4.88***	4.22***
SVM	2.51***	4.26***	1.75***

***0.001; **0.01; *0.05

To evaluate the relative significance of each feature using the random forest classifier, we conducted a feature importance analysis as shown in Table 4. The importance score is normalized by the total importance scores in each model to show each feature’s relative importance in the prediction of each model. In Model 1, age and length of hospitalization contribute the most to the classification. Also, sociomarkers contributed more than the race variable, which is known as an important factor in the previous study.^29^ Among the sociomarkers, the blight prevalence and neighborhood quality are the most critical features and they are as important as gender. In Model 2, the length of hospitalization is the most critical feature in prediction. In Model 3, the age feature is the most important feature and gender and neighborhood inequality are next.

**Table 4:** Feature importance results obtained from RF

	All-inclusive model (Model 1)	Biomarker (Model 2)	Sociomarker (Model 3)
Age	0.22	0.26	0.44
Gender	0.05	0.04	0.08
Race	0.02	0.03	0.04
Duration	0.34	0.61	NA
Severity	0.07	0.05	NA
Blight	0.05	NA	0.07
Broken window	0.04	NA	0.05
Dumping trash	0.04	NA	0.06
Neighborhood quality	0.05	NA	0.06
Neighborhood inequality	0.04	NA	0.08
Poverty	0.05	NA	0.06
Medicaid	0.03	NA	0.06

## Discussion

Our results suggest that sociomarkers in Memphis study area aggregated on the ZIP code level can be reliable predictors of pediatric asthma patients at risk of hospital revisit within a year. Although we created the most well-performing model using all data including demographic features, biomarkers, and sociomarkers, the accuracy of the sociomarker-based model alone is still 61%. Although, the biomarker-based model still more accurately predicts the patients at risk, incorporating socio-markers to the predictive model significantly improves the accuracy by 0.65% with the RF classifier and by 2.51% with the SVM classifier. Our findings are in accordance with the widely accepted theory of non-genetic components of health outcomes: 15% contributed by social circumstances, 5% by environmental exposure, 10% by health care, and 40% by behavioral patterns; the balance (30%) is believed to be contributed by genetic predisposition.^12^ Sociomarker-based model alone can predict the patient at risk with the accuracy of 61%. Without knowing any information directly related to the symptom-related conditions, a model only based on social and neighborhood conditions can predict two out of three patients at risk of hospital revisits correctly. Furthermore, our findings show that environmental conditions play a more critical role in prediction compared to the socio-economic status of the patients.

However, the inference of our findings is constrained by three limitations. The first source of bias comes from the time frame: we only use a 12-month period of observation in 2016. Therefore, revisits beyond the observation window are not considered. The results must be interpreted based on the time frame condition and expanding the observation window can improve the accuracy of prediction. Secondly, the patients may have visited different hospitals which are not captured in the data analyzed here because the authors utilize the medical record from one hospital only. Lastly, the definition of a neighborhood we are using in this study is an area of an entire ZIP code. ZIP code level may not have the fine granularity required to capture detailed social gradients, and smaller geographical boundaries, such as census tract level, may yield more accurate prediction.^15,22^ Unfortunately, the most detailed residential information we can access is the ZIP code level. Although a ZIP-code may not be the most fine-tuned boundary capturing neighborhood conditions, fortunately, ZIP code level data perform well in our study, given the low density in the subject area. Despite these limitations in data, which can be challenging for the machine learning-based classification process, our models perform stably across training and test sets.

This study provides two distinctive contributions. First, by employing machine learning-based classification models, we systematically and empirically proved that sociomarkers can predict health outcomes at the individual level in pediatric asthma cases with 61% of accuracy. We unpack the relative importance of social features in pediatric asthma hospital revisits. Second, by merging multiple data sources with detailed neighborhood level and social data, we directly show the importance of living environment and social conditions for their contributions to individual health outcomes. The Property Hub dataset provides a unique research opportunity to explore the link between detailed neighborhood/built environment qualities and health outcomes. In addition to the socio-economic status, future studies for pediatric asthma readmission require to rigorously consider environmental conditions as well.

Bringing sociomarker features into the health surveillance system may improve decision-making and detection of at-risk patients for hospital revisit. Understanding of the pathway as to how social inequalities are channeled to health inequalities is a matter of supreme import, and the neighborhood is the key unit for the mechanism. Detailed neighborhood-level data can help us to unpack the pathways of social inequalities to health disparities(see Mahajan et al.^44^ for example) and allow the improvement of public health through comprehensive surveillance systems. This has important policy implications since our model suggests a cost-effective surveillance method. The features included in the sociomarker predictive model, which do not require further data mining, nor collecting additional data beyond the data collection practiced in a daily medical routine, are relatively simple, and therefore cost-effective.

## Methods

### Dataset

In this paper, we integrated data collected from three different sources: pediatric asthma encounter records collected from 1 January to 31 December 2016, at a children’s hospital; the 2010 U.S. census data; and the housing and neighborhood quality survey data collected by the Memphis Property Hub. The patient’s medical data are collected from the 255-bed Le Bonheur Children’s Hospital located in Memphis, TN. This study has been reviewed and approved by the University of Tennessee Health Science Center Institutional Review Board, and waiver of patient consent was granted for the retrospective study. From the U.S. Census data, we determined the proportion of individuals living under the federal poverty line. The Property Hub data provided detailed neighborhood quality data. After merging these data sets at the ZIP code level, we examined the effect of social features in identifying the group of patients who visited the hospital more than once during the observation period. We only used the first-time visit during the observation period (3678 cases) to avoid over-counting of the same patients. For social features, the proportion of individuals living below the federal poverty level, blight prevalence, housing quality, neighborhood inequality, trash presence, and the broken window prevalence within the ZIP code area of patients’ residences were included. Additionally, the model contained demographic features such as age, gender, insurance type, and race (African American and White). The descriptive statistics of all variables included in the study are presented in Table 1.

### Data analysis

The objective of this paper is to analyze the effect of using features related to social, demographic, and symptom parameters in predicting hospital revisit of pediatric asthma patients within one year of the initial intervention at the hospital. We, therefore, analyzed three models in different studies to evaluate the efficacy in predicting hospital revisit. In the all-inclusive model (Model 1), we used all 12 features including demographic attributes, biomarkers, and sociomarkers, and all sub-variables are listed in Table 1. In the biomarker-based model (Model 2), we use 5 predictive variables of demographic attributes and biomarkers. Lastly, in the sociomarker-based model, we include 10 variables of demographic attributes and sociomarkers. We have used the normalized value for broken windows, dumping prevalence, and unoccupied houses in the ZIP code. In all models, to examine how sociomarkers perform in detecting the patient at risk of a hospital revisit, we implemented a random forest-based classification model.

A random forest is a supervised classification approach which combines the results of many decision trees to reduce overfitting and improve generalizability.^38^ In this paper, we have used an ensemble of 30 decision trees. The outcome variable of the machine learning-based classification model was: Class 0 if the patient visits the hospital within the year of our study, or visits only one time; or Class 1 if the patient revisits (more than 1 visit). Among 3678 unique patients in the dataset, there are only 823 patients in Class 1. Therefore, to overcome the class imbalance issue, we have used 823 patients’ data from each class. Further, to avoid overfitting and ensure generalizability, we split the dataset (without replacement) as training and test with a proportion of 80 and 20%, respectively. We implemented a five-fold cross-validation on the 80% training set by dividing it into five distinct folds. Five machine learning-based classification models were then built using four-folds of data and tested on the remaining one-fold. This process provided the cross-validation predictions for 80% training data. An ensemble of these five models was further implemented on the 20% test set to control and avoid overfitting. To avoid the sampling bias due to the selection of 823 cases from Class 0, we repeat this overall process 1,000 times by randomly selecting 823 cases from Class 0 and present the average performance over 1000 iterations.

Since the ZIP code characteristics are stationary for every observation within the same ZIP code, we paid an extra attention to Model 3, which is using only sociomarkers as predictors, to ensure generalizability of our models across ZIP codes. We applied the similar cross-validation methodology for Model 3 as described above, by randomly selecting 80% ZIP codes (i.e., 23 out of 29) to construct the training set and remaining 20% (6 of 29) ZIP codes in the test data. Using the observation from training ZIP codes, we built a predictive model and tested it on the remaining observations from 6 testing ZIP codes. We repeated the sampling process 1000 times to avoid sampling bias and reported average classification statistics obtained from five-fold cross-validation on the training dataset and test dataset.

We also compared results with a non-linear SVM classifier using the same set of features in Model 3. A SVM model was generated with a Gaussian radial basis function kernel with the kernel scale of 0.83.41 The parameters used for training RF and SVM models, i.e., the number of decision trees, ensemble method, kernel function, etc. were obtained empirically. We compared results of SVM and random forest-based classifier for both training and test sets for each of these studies. All analysis was performed in MATLAB, 2017b version.

### Code availability

All unique code and algorithms generated for data analysis are available upon reasonable request.

## Data Availability

The de-identified datasets can be provided upon request.
